# Accelerating Automated Stomata Analysis Through Simplified Sample Collection and Imaging Techniques

**DOI:** 10.3389/fpls.2020.580389

**Published:** 2020-09-25

**Authors:** Luke Millstead, Hiranya Jayakody, Harsh Patel, Vihaan Kaura, Paul R. Petrie, Florence Tomasetig, Mark Whitty

**Affiliations:** ^1^School of Mechanical and Manufacturing Engineering, University of New South Wales, Sydney, NSW, Australia; ^2^Crop Sciences Division, South Australian Research and Development Institute, Waite Campus, Urrbrae, SA, Australia; ^3^Mark Wainwright Analytical Centre, University of New South Wales, Sydney, NSW, Australia

**Keywords:** stomata analysis pipeline, stomata sample collection, stomata pore measurement, high-throughput analysis, microscope imagery

## Abstract

Digital image processing is commonly used in plant health and growth analysis, aiming to improve research efficiency and repeatability. One focus is analysing the morphology of stomata, with the aim to better understand the regulation of gas exchange, its link to photosynthesis and water use and how they are influenced by climatic conditions. Despite the key role played by these cells, their microscopic analysis is largely manual, requiring intricate sample collection, laborious microscope application and the manual operation of a graphical user interface to identify and measure stomata. This research proposes a simple, end-to-end solution which enables automatic analysis of stomata by introducing key changes to imaging techniques, stomata detection as well as stomatal pore area calculation. An optimal procedure was developed for sample collection and imaging by investigating the suitability of using an automatic microscope slide scanner to image nail polish imprints. The use of the slide scanner allows the rapid collection of high-quality images from entire samples with minimal manual effort. A convolutional neural network was used to automatically detect stomata in the input image, achieving average precision, recall and F-score values of 0.79, 0.85, and 0.82 across four plant species. A novel binary segmentation and stomatal cross section analysis method is developed to estimate the pore boundary and calculate the associated area. The pore estimation algorithm correctly identifies stomata pores 73.72% of the time. Ultimately, this research presents a fast and simplified method of stomatal assay generation requiring minimal human intervention, enhancing the speed of acquiring plant health information.

## Introduction

The size and density of stomata have been studied as important plants traits since the early 19^th^ century (Banks, 1805). Stomata pores, located on the plant leaf epidermis, play a major role in regulating the diffusion for both carbon dioxide and water (Dow et al., 2014) and their distribution provides important information about plant developmental biology (Lau and Bergmann, 2012). Recent works suggest that stomatal closure under water stress could result in vein embolism, which can cause the plant water transport system to collapse (Brodribb et al., 2016). Hence, stomata shape and behaviour are identified as direct indicators of plant health and the surrounding environmental conditions (Beerling and Chaloner 1993a; Beerling and Chaloner 1993b; Sadras et al., 2012).

Analysis of stomata is also an important aspect of paleoecology; for example, stomatal index (i.e. the ratio between the number of stomata and epidermal cells) of fossil plant cuticles can provide valuable insights into the atmospheric carbon dioxide levels in a given era (Beerling and Chaloner 1993b; Beerling and Royer, 2002). In addition, the undulation index (waviness of stomata cell wall), which is physiologically affected by light, correlates well with growing degree-days (GDD), which provides information on seasonal change (Smith et al., 2010; Wagner-Cremer et al., 2010; Wagner-Cremer and Lotter, 2011) in a given period of time. Thus, microscope analysis of stomata plays a major role in present day agriculture as well as modelling climate change over long periods of time.

Stomatal aperture is often measured using the microscope imaging of leaf samples, epidermal peels, or imprints (Dow et al., 2014; Eisele et al., 2016; Jayakody et al., 2017). These images are analyzed using image processing software such as ImageJ (Rasband, 1997), which enables manual measurements to be made on a computer interface. The manual measurement of stomata is sufficient when it is only necessary to measure a small number; however, this would prove unsuitable when processing an entire leaf surface. For the analysis of larger leaf areas, automated image processing techniques are required.

One of the first papers to implement digital image processing for automating stomatal measurements was Omasa and Onoe’s (Omasa and Onoe, 1984) work with stomatal aperture. Here, the authors applied a Hanning Filter, discrete inverse Fourier transform and thresholding to measure individual stomata. In the decades following this paper, numerous advancements have been made in computer vision and microscopy. These advancements have supported improvements in the automation of stomatal analysis. Whether it be through detecting the unique fluorescence emission of stomatal guard cells under UV excitation (Karabourniotis et al., 2001), through rhodamine 6G staining (Eisele et al., 2016), or through template matching (Laga et al., 2014), it has ultimately been the automatic measurement, not detection, of stomatal pores in large samples that has proven most difficult. More recent research (Jayakody et al., 2017; Toda et al., 2018; Fetter et al., 2019; Sakoda et al., 2019) utilizes machine learning and image processing to detect stomata (and sometimes classify the state of the stomata) in microscope images. The accuracy levels achieved in these studies shows promise and enables plant scientists to conduct high-throughput analysis for stomata detection. However, once stomata are detected, correctly measuring the stomatal pores requires additional image processing steps (and sometimes human intervention), which can increase the overall processing time. Thus, it is important to build algorithms which go beyond stomata detection, and reliably measure pore opening under varying image quality.

Although machine learning can enable high-throughput microscope image analysis, the efficiency of the overall pipeline still depends on sample collection and imaging techniques. Hence, for current image processing techniques to have any practical value in the field, microscope samples must be collected and imaged quickly, accurately and in sufficient detail to measure stomatal pore areas. Most current sample collection processes produce images with suitable quality for digital image processing. However, many of these techniques require complex chemicals or intricate leaf manipulations, which are often time consuming and impractical for use in the field by untrained operators (Weyers and Travis, 1981; Celine et al., 2012; Eisele et al., 2016; Monda et al., 2016; Yuan et al., 2020).

There are two common methods that are used to collect samples quickly and simply. The first is the silicon impression method, described by Weyers and Johansen (1985), which uses dental resin to create a negative impression of the leaf surface, and nail varnish to transfer this imprint onto a microscope slide. The second method replaces the dental resin with nail polish, so that a direct impression is made. Upon drying, adhesive tape is used to transfer the imprint to a microscope slide (Rogiers et al., 2011). These methods result in samples of reasonable quality which are suitable for automated stomata detection.

One of the major bottlenecks in this process is the time taken to image the collected samples. As such, techniques used to obtain microscope images from samples must be re-examined to develop a streamlined yet accurate process. Currently, many researchers use simple light-field or other manual stage microscopes to obtain their results (Jayakody et al., 2017; Fetter et al., 2019), which is sufficient to examine small numbers of stomata. However, if a larger portion of the leaf is to be covered, the manual stage movements can take several hours, even with a motorized stage. Additionally, the large proportion of veins create protrusions in the epidermal surface, which requires refocusing the microscope upon every movement of the stage. Consequently, most current research on stomate detection and analysis relies on input images containing up to 40 stomata at most (Li et al., 2019), making it difficult to measure density or observe patterns across a leaf. With these limitations apparent, it is important to investigate fast imaging methods which require minimal manual effort.

We present an accelerated end-to-end process to identify and measure stomata, whilst significantly reducing the manual labour requirements. Two simple approaches for sample collection were assessed, with the aim of producing high quality samples for imaging. Then a microscope slide scanner was utilized to rapidly image the samples, eliminating the need for manual staging and focusing of the sample. A Convolutional Neural Network was implemented to detect stomata from the feature rich images generated by the slide scanner and a novel stomatal pore measurement algorithm is proposed to identify the pore area regardless of the colour intensity of the pore. This is a streamlined solution for efficiently analysing stomatal morphology, distribution, and patterning across large leaf surfaces.

## Materials and Methods

### Simplified Sample Collection

One of the primary aims of this paper is to determine the most effective sampling technique which is both simple and reliable. The common nail polish imprint method (Miller and Ashby, 1968) meets both these requirements due to its simplicity. The steps involved in the common nail polish imprint method are as follows:

1. A thin layer of nail polish (Revlon Ultra, Revlon Consumer Products Corporation, New York, NY, USA was used in this research) is applied to the abaxial surface of the leaf. The surface covered by the nail polish is set to approximately 30 mm in length and 9 mm in width and is selected such that major veins are avoided.
2. The nail polish is then left to dry for approximately five minutes.
3. One piece of clear adhesive tape is pressed onto the dry nail polish.
4. The tape is removed from the leaf surface and the adhesive side secured to a plastic sleeve for transport to a laboratory environment.

**Figure 1:**
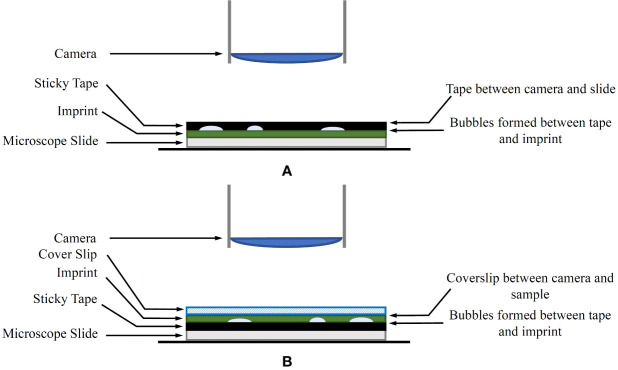
**(A)** The original imprint method. The sticky tape is between the camera and the sample. **(B)** The modified imprint method. The sticky tape and bubbles are no longer between the microscope and the sample. Instead, an appropriate coverslip is covering the sample.

A common issue with the traditional leaf imprint method is the introduction of air bubbles when securing the imprint to the microscope slide using tape. The tape also tends to deform to the shape of the uneven leaf surface, which may create focusing issues during the imaging process. A modified approach is proposed for mounting the imprint on the microscope slide to combat this as follows:

1. In the laboratory, the tape is removed from the plastic sleeve, transferred to a thin glass coverslip and the adhesive side is pressed down to ensure a flat surface.
2. The top (non-adhesive) surface of the tape, including the attached coverslip, is mounted on a microscope slide using transparent sticky tape on the corners of the coverslip as shown in **Figure 1B**. Optionally, a product similar to Vectashield mounting medium (Vector Laboratories, Inc. Burlingame, CA, USA) can be used to mount the coverslip onto the microscope slide. If a mounting medium is used, several glass weights should be placed on the coverslip to distribute the mounting medium evenly.

A diagram of the modified method in comparison to the original technique is shown in **Figure 1**. The coverslip aims to reduce any unevenness of the leaf imprint, allowing shallow depth-of-field sensors to keep larger areas of the sample in focus. With this approach, any artefacts generated on the adhesive side of the tape are no longer obstructing the view of the microscope.

Samples from four plant species, Vitis vinifera L. x V. rupestris Scheele ‘Ganzin Glory’, Prunus armeniaca ‘Moorpark’, Citrus sinensis L. Osbeck ‘Valencia’, and Vinca major L. ‘Periwinkle’, were collected from Belair, Adelaide. The samples were prepared using both traditional and modified mounting approaches for comparison purposes. A detailed comparison between the proposed sampling method and the common nail polish imprint method is presented in *Simplified Sample Collection*.

### Microscope Slide Scanner for Imaging Samples

A manual-stage optical microscope is not capable of capturing a complete 30 mm × 9 mm leaf sample with a single image. Instead, the sample must be moved, and the microscope refocused prior to capturing each image. Once multiple images are captured covering the sample, they need to be stitched together to create a single image of the leaf.

These issues are solved by using a microscope slide scanner. Used in the field of cell pathology, slide scanners can rapidly produce high-quality images of the complete sample slide at once. This is achieved by an automated process where the slide is carefully moved under a line-scan camera. The lines are then automatically stitched together to produce a single high-resolution image of the complete sample. Imaging the complete slide at once allows researchers to gain a better understanding on macro level characteristics such as stomata patchiness. In addition to speeding up the image capture process, another major advantage of the slide scanner is its ability to store multiple microscope slides in the device. This feature allows users to load many samples and image them in a single run without adjusting settings for each new sample. In this work, an Aperio^®^ XT (40x) brightfield slide scanner (Wetzlar, Germany) is used. The device uses linescan technology to generate images at a resolution of 0.25 um/pixel and holds up to 120 slides at one time. The performance of the slide scanner is compared with a manual stage optical microscope in *Imaging With Microscope Slide Scanner*.

### Stomata Detection With a Convolutional Neural Network

Stomatal pore area measurements require the identification of stomata in a microscope image (Dow et al., 2014); with a small number of stomata, this can be achieved using manual image analysis tools. More recently, higher order image processing and machine learning has been used to automate this process (Laga et al., 2014; Liu et al., 2016; Jayakody et al., 2017; Toda et al., 2018; Fetter et al., 2019; Sakoda et al., 2019). In this work, a CNN (Lecun et al., 1998) based on the MATLAB® implementation of the AlexNet (Krizhevsky et al., 2012) network was used to identify stomata (MathWorks, 2018). AlexNet is pre-trained on more than one million images and can facilitate transfer learning, which takes the pretrained network and utilizes its feature extraction capabilities as a starting point to learn new detection tasks. This requires fewer training images, which reduces the time required to automate the overall process for a new image target. The process of stomate detection using AlexNet is described below. The training data for transfer learning comprised of images collected through both traditional and modified sample collection methods.

**Figure 2:**
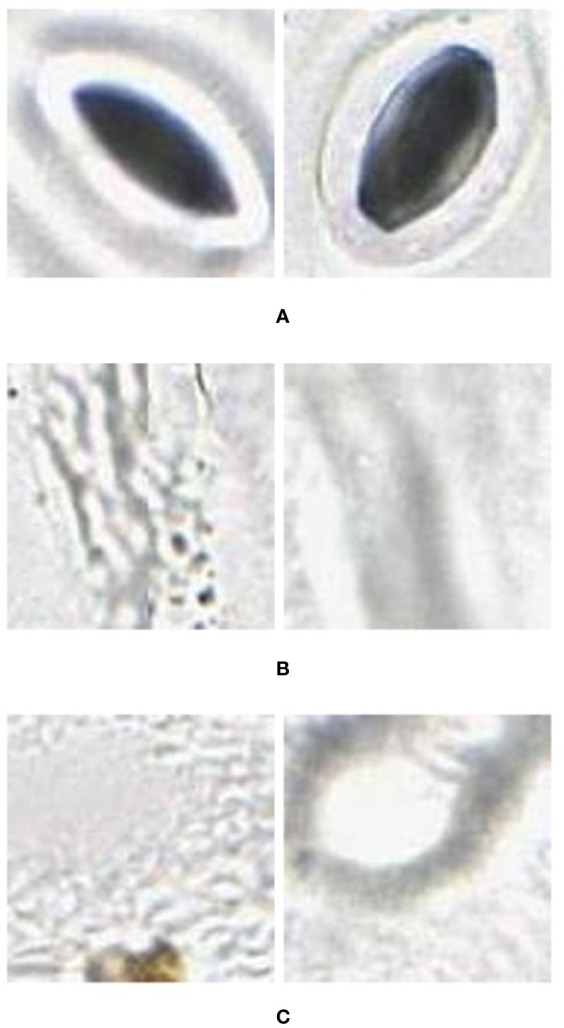
Example **(A)** stomata (positive), and **(B)** veins and **(C)** background (negative) used to train the AlexNet neural network. These were extracted from images (in regions separate to that being classified) collected using the modified imprint method and imaged with the slide slidescanner.

1. Using the images extracted for training, a training set was prepared with images assigned to three categories: stomata, vein, and background (as shown in **Figure 2**).
2. Feature vectors are extracted from the training data to train a classifier using AlexNet. Particularly, AlexNet is eight layers deep (MathWorks, 2018) and, when used for feature extraction, the neural network is terminated at one of the fully connected middle layers. This layer outputs the feature vector representing the activations for the input images.
3. Using MATLAB’s Classification Learner application (Machine Learning toolbox), a quadratic Support Vector Machine classifier is trained with the feature vector.
4. The classifier is then applied to the image through a classification window of predefined size, translated across the image by sliding the window. This produces a mask of the image indicating the location of each stomate.
5. To calculate precision, recall, and accuracy, the stomata are manually labelled using a custom GUI. By discretising the manually and automatically labelled images and comparing each grid value (1 if stomate, 0 if background), the number of false positives (FP), true positives (TP), false negatives (FN) and true negatives (TN) were determined.

In order to reduce the processing time involved with applying a sliding window across the entire image, the program first splits the slide scanner image into smaller tiles which are processed individually, and then reassembles the labelled results into a complete image.

### Stomata Pore Area Calculation

**Figure 3:**
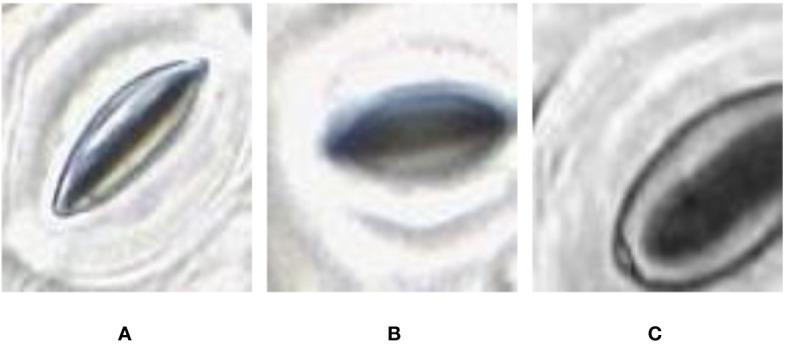
Quality variation of the stomata captured at different parts of the microscope image. **(A)** sharp image. **(B)** blurry image **(C)** partially captured image.

With the methods proposed in *Microscope Slide Scanner for Imaging Samples*, the slide scanner is able to produce feature-rich images where stomata can be clearly identified. However, the sharpness of the image can slightly vary along the image due to the uneven nature of the leaf surface, causing variation in focus. This variation directly affects the quality of each individual stomate image based on their location on the leaf. This results in stomata images with different image qualities (**Figure 3**).

**Figure 4:**
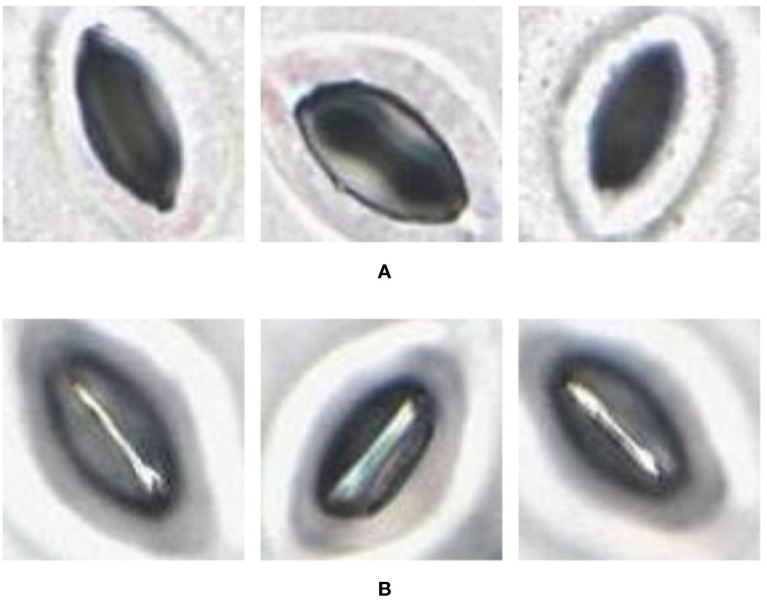
Pore area captured under different lighting conditions. **(A)** Pore area is dark compared to the surroundings. **(B)** Pore area has a lighter colour compared to the surroundings.

In some of the stomata images captured by the slide scanner, the pore area appears darker than the surrounding guard cells whereas in other stomata images the pore area appears lighter than the guard cells (**Figure 4**). These variations depend largely on focus; due to significant variation in height across the sample, and the lens’ single plane of focus, regions can appear either in or out of focus. The lighter stomata, for example, are in focus, with the focal plane located in the middle of the pore. When the focal plane is situated slightly above the middle, the reflection of light bouncing off the guard cells results in darker stomata pores. This requires the pore estimation algorithm to be robust against variations in colour space. Existing algorithms require stomata colour space to be consistent and are often tuned to a specific plant species (Karabourniotis et al., 2001; Laga et al., 2014; Jayakody et al., 2017), thus making them unsuitable to analyze images from the slide scanner.

To achieve this goal, a novel approach based on stomata cross section analysis and binary segmentation is proposed. Prior to developing the pore estimation algorithm, the following assumptions were made regarding the stomatal pores.

1. The centre of the stomatal pore is located reasonably close to the centre of the bounding box containing the stomate. This bounding box is generated by the CNN proposed in *Stomata Detection With a Convolutional Neural Network*. This assumption allows the algorithm to reject stomata-like shapes located at the edges of a bounding box.
2. A stomata area is always larger than a predefined value. In this case, a stomate is assumed to be larger than 50 pixels^2^ in area. This allows the algorithm to reject detections resulting from dust particles and air-bubbles.

The pore estimation algorithm consists of the following steps.

**Figure 5:**
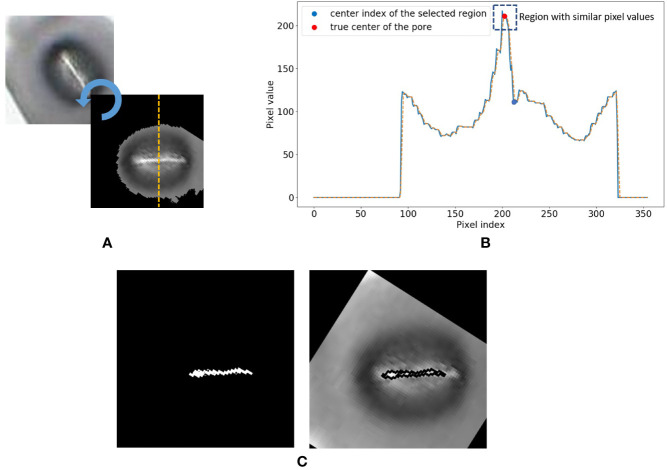
Stomate cross section analysis. **(A)** Horizontal alignment of the stomate. **(B)** Identification of true center of the pore using peak/valley detection. **(C)** Final result.

1. Contrast Limited Adaptive Histogram Equalisation (CLAHE) is applied to the original input image.
2. The contrast and sharpness of the CLAHE image is improved.
3. The CLAHE image is converted to a grayscale image.
4. The Grayscale image is converted to a binary image via Otsu’s thresholding.
5. The binary image contains multiple regions. Regions with areas no larger than a predefined size are removed from the image.
6. The largest region closest to the centre of the image is selected, and all other regions are removed from the image. This region is selected as the mask which represents the stomate.
7. The mask is then applied to the grayscale image in Step 3. Rotate the image using the major axis orientation of the mask. Now the area containing the stomata is aligned horizontally in the image.
8. Now consider the vertical cross-section which goes through the centroid coordinate of the mask as shown in **Figure 5A**. The intensity values of the pixels which lie along this cross-section line can be plotted as shown in **Figure 5B**. The following steps are adopted to find the pore area of the stomate.
  - Identify all the valleys and peaks on the cross-section plot.
  - Identify the valley or peak closest to the centroid pixel. This valley or peak is the centre of the stomatal pore (See **Figure 5B**).
  - If the coordinate corresponding to the stomatal pore centre is a peak, the pore area is lighter than the surrounding region, and if the index corresponding to the stomatal pore centre is a valley, the pore area is darker than the surrounding region.
  - Once this pore centre is identified, select all pixels of which the intensity values are similar to that of the pore centre, and also connected to the pore centre pixel (dotted box on **Figure 5B**).
  - This connected set of pixels represent the pore region of the stomate (See **Figure 5C**).

**Figure 6:**
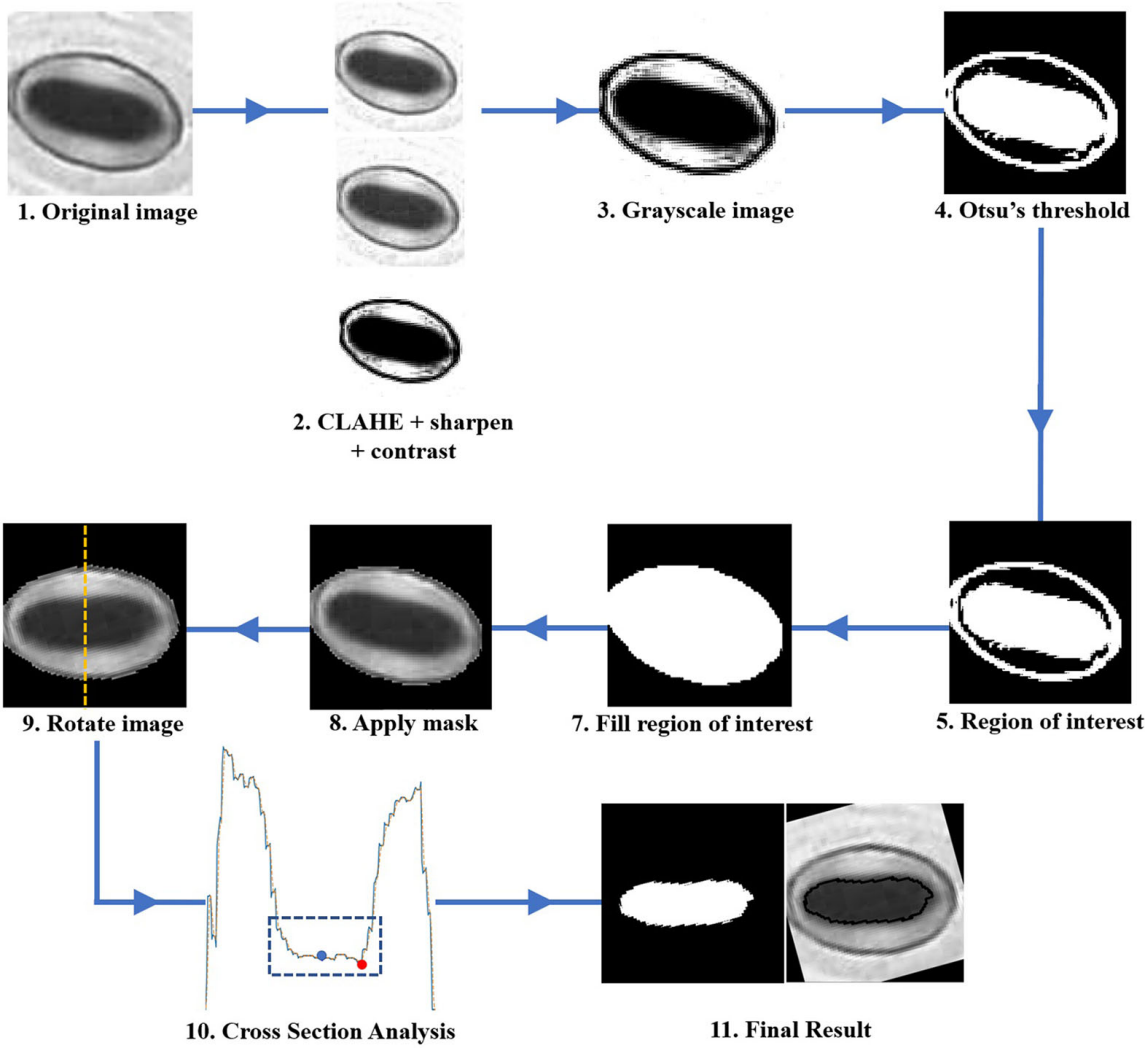
Flowchart describing the step-by-step approach of the pore area calculation algorithm.

The step-by-step approach of the algorithm is shown in **Figure 6**. The performance of the proposed pore estimation algorithm is discussed in detail in *Pore Area Estimation*.

## Results

### Simplified Sample Collection

This section focuses on evaluating the performance of the modified nail polish imprint method compared to the traditional method, including their suitability for use with the proposed slide scanner technique. In these comparisons, special attention is given to the time taken in preparing the samples as well as the quality of the resulting images.

Securing the imprint on a plastic sleeve and transporting it to a laboratory environment before mounting onto a slide did not have any discernible negative impact on the sample quality. This approach also reduced contamination and the sample collection time in the field. Additionally, when waiting for the nail polish on one sample to dry, it was efficient to apply the polish to additional leaves in a parallel fashion. Using these methods, it was possible to collect a sample every 2 min.

**Figure 7:**
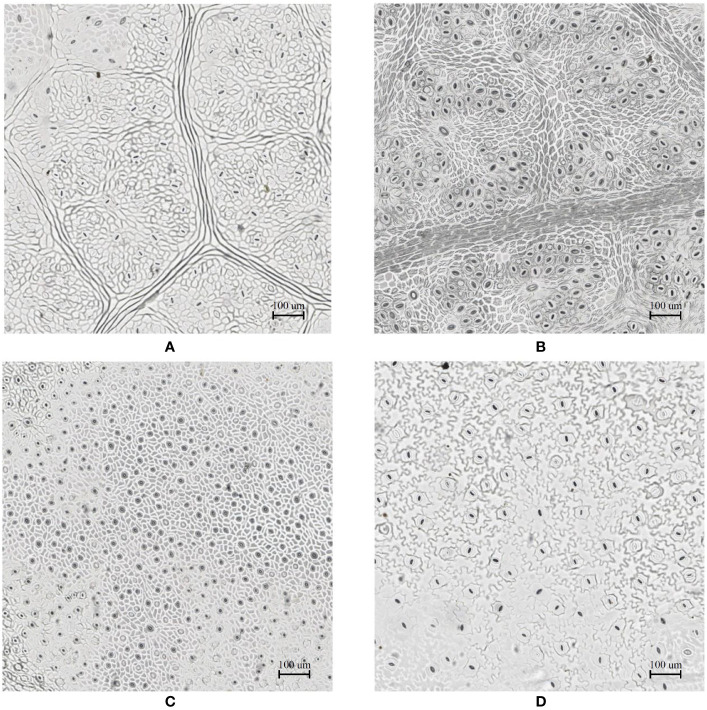
Slide scanner images generated by capturing a section of the samples collected using the modified nail polish imprint method. **(A)** Vitis vinifera L. x V. rupestris Scheele ‘Ganzin Glory’. **(B)** Prunus armeniaca ‘Moorpark’. **(C)** Citrus sinensis L. Osbeck ‘Valencia’. **(D)** Vinca major L. ‘Periwinkle’.

**Figure 8:**
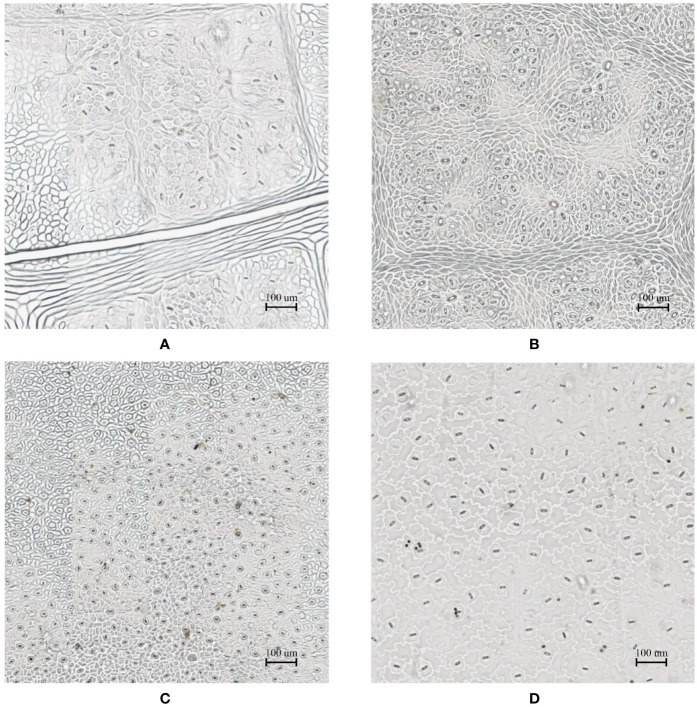
Slide scanner images generated by capturing a section of the samples collected using the traditional nail polish imprint method. **(A)** Vitis vinifera L. x V. rupestris Scheele ‘Ganzin Glory’. **(B)** Prunus armeniaca ‘Moorpark’. **(C)** Citrus sinensis L. Osbeck ‘Valencia’. **(D)** Vinca major L. ‘Periwinkle’.

In the laboratory, the traditional approach of securing the sample directly to a microscope slide took 2.5 min on average. Using the modified nail polish imprint method, the average slide preparation time was measured at 3.5 min. **Figures 7** and **8** indicate that both the traditional and modified sample collection methods produce high quality samples when imaged using the slide scanner technique. For each of the four species, stomatal pores are clearly discernible from background epidermal cells.

Interestingly, no significant difference in quality between the modified and traditional techniques could be observed from our results. This suggests that the quality of the result is dominated more by the sample collection process on-site rather than the mounting technique in the laboratory. Based on the initial assumptions behind the modified mounting method the effect of the modified approach may only become significant if the samples are collected under difficult conditions and contain bubbles or other debris.

### Imaging With Microscope Slide Scanner

To measure the performance improvement introduced through the proposed imaging technique, 40 samples were imaged using the Aperio^®^ XT (40×) brightfield slide scanner (Wetzlar, Germany). The imaging time and quality was then compared with the Olympus Olympus^®^ BX53 manual stage microscope. A summary of the processing times of the slide scanner in comparison to the manual stage microscope is presented in **Table 1**.

**Table 1:** Comparison of sample collection, preparation and imaging techniques trialled.

	Original nail polish imprint method with slide scanner	Modified nail polish imprint method with slide scanner	Original nail polish imprint method with manual microscope
Sample collection time	2 min	2 min	2 min
Sample preparation time	2.5 min	3.5 min	2.5 min
Time taken to image section of sample	10 min	10 min	120 min
Time taken to image entire sample	0.5–0.66 h	0.5–0.66 h	120–140 h (estimate)

For sample collection and preparation, the original and modified nail polish methods display similar results for sampling effort, time required and resultant image quality. For imaging, the slide scanner technique offers significant savings in manual effort and imaging time when compared to traditional manual stage microscopes.

The slide scanner required 15 min of set-up and 10 min to capture a selected region of the sample. The setup process consists of loading the slides in the device and selecting the focus points. Multiple focus points were selected to ensure the stomata remained in focus despite the variation in sample height. The slide scanner allows the coverage of the sample to be scaled up with a minimal increase in processing time. An entire sample of size 30 mm × 9 mm, for example, can be imaged in 30–40 min. Since multiple slides can be loaded on the device (120 glass slides for this model), the device was able to image all 40 samples with a single set-up.

Comparatively, it took two hours to image a 4 mm × 1 mm leaf imprint image using the Olympus^®^ BX53 manual optical microscope. To cover an area the size of 30mm × 9mm, over 70 images are required. If the images need to be stitched together to analyze macro level patterns, images should be captured with some overlap, driving up the number of total images required as a result. Due to the uneven surface of the imprint, each image needed to be focused separately. As the manual-stage microscope is only able to capture a small portion of the leaf surface at a time, special attention was given to ensure overlapping between adjacent images so that the images can be stitched together to form the final leaf surface. The slide scanner approach is clearly the faster approach, with over 100× time improvement over manual processes.

**Figure 9:**
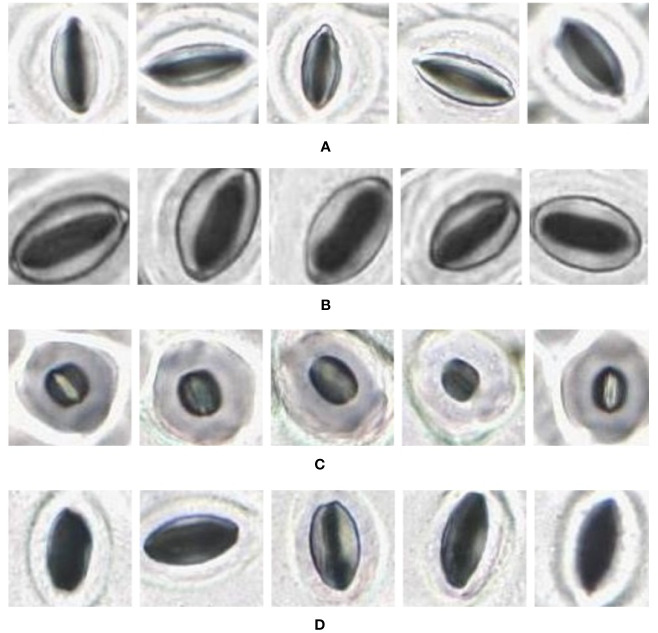
Individual stomata captured from modified samples using the slide scanner. Guard cell boundaries and background epidermal cells are clearly visible. **(A)** Vitis vinifera L. x V. rupestris Scheele ‘Ganzin Glory’. **(B)** Prunus armeniaca ‘Moorpark’. **(C)** Citrus sinensis L. Osbeck ‘Valencia’. **(D)** Vinca major L. ‘Periwinkle’.

**Figure 10:**
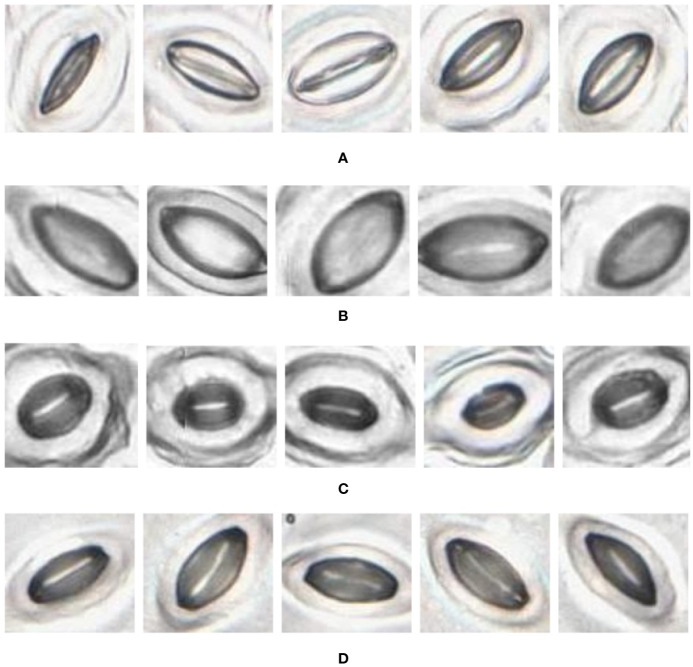
Individual stomata captured from traditional samples using the slide scanner. Guard cell boundaries and background epidermal cells are clearly visible. **(A)** Vitis vinifera L. x V. rupestris Scheele ‘Ganzin Glory’. **(B)** Prunus armeniaca ‘Moorpark’. **(C)** Citrus sinensis L. Osbeck ‘Valencia’. **(D)** Vinca major L. ‘Periwinkle’.

The slide scanner produced feature rich images, suitable for stomata detection and pore measurement. The edges of stomatal guard cells and the presence of background epidermal cells are well defined in the slide scanner images (**Figures 9**, **10**). Whilst blurred sections at various locations across the sample were observed due to the varying distance to the lens from the sample, the image contained plenty of regions with little to no blur containing more than 1,000 stomata, allowing users to observe patterns spanning across large areas of the sample. The optical microscope and the slide scanner produce images of similar quality; but the slide scanner dramatically improves the speed and area that can be imaged.

### Stomata Detection Using Convolutional Neural Networks

The neural network created using AlexNet transfer learning was run on slide scanner images of samples collected using the traditional and modified method, for each of the four species; this amounted to eight separate images, including 4,986 stomata in total. Each image included stomata that were both in and out of focus.

The results of running the classifier on each image can be seen in **Table 2**. The overall F-score was 0.817, with the highest of 0.897 recorded for the Citrus sinensis L. Osbeck sample collected using the modified technique; this maximum value was achieved for an image containing 1,168 stomata. The average precision and recall were 0.778 and 0.865, respectively. These results look promising, and provide evidence of the classifier’s ability to adequately identify stomata in a background dense with similar features (**Table 2**).

**Table 2:** Results of AlexNet Neural Network classifier applied to 6 images.

Species	Collection technique	Number of stomata	Precision	Recall	F-score
**Vitis vinifera L. x V. rupestris Scheele**	**Traditional**	248	0.6692	0.9254	0.77672
	**Modified**	207	0.7825	0.9055	0.83952
**Prunus armeniaca**	**Traditional**	791	0.7291	0.7388	0.73392
	**Modified**	917	0.7265	0.7517	0.73889
**Citrus sinensis L. Osbeck**	**Traditional**	932	0.7290	0.9076	0.80855
	**Modified**	1168	0.9089	0.8850	0.89679
**Vinca major L**.	**Traditional**	406	0.8673	0.9095	0.88790
	**Modified**	317	0.8138	0.8943	0.85215

A number of false positives were incorrectly identified as stomata in the images. Some of these features appeared similar to a stomatal pore that is lighter than its surroundings. These may indicate a stomate that has not been reproduced correctly by the imprint or is completely closed, or possibly an elliptical epidermal cell. Similarly, some false positives closely resemble a stomatal pore that is darker than its surroundings. Again, this appears to be a feature of the background reproduced by the sampling technique and slide scanner. Finally, random elliptical features in the background were captured by the high-detail slide scanner. Such false positives can potentially be eliminated with further training samples. In general, it is the precision that constrains the F-score achieved by the classifier, primarily due to the presence of false positives arising from the high level of detail in the slide scanner images. However, these false positives proved to be generally harmless as they were eliminated in the pore identification software, as having no discernible pore to measure.

The false negatives in the results can be attributed to the wide variety of stomata captured by the high level of detail in the slide scanner images. False negatives can be reduced, and the recall can be increased by collecting more training samples and ensuring a standardized method of sample collection. The CNN approach to stomate detection is suitable for the slide scanner images and future investigations are recommended to further optimize this method.

### Pore Area Estimation

The pore estimation algorithm was developed using OpenCV 3.4 in Python 3.6. The algorithm was applied to 1,706 individual stomata generated by the CNN classifier over the traditional and modified samples collected from the four plant species. The results generated were analyzed manually for erroneous pore estimations (**Table 3**).

**Table 3:** Results of pore area estimation.

Species	Collection technique	Total Images	No detections	Available	Incorrect	Correct	Percentage correct
**Vitis vinifera L. x V. rupestris Scheele**	**Traditional**	200	21	179	66	113	63.13%
	**Modified**	200	4	196	43	153	78.06%
**Prunus armeniaca**	**Traditional**	177	28	149	32	117	78.52%
	**Modified**	200	29	171	17	154	90.06%
**Citrus sinensis L. Osbeck**	**Traditional**	200	20	180	40	140	77.77%
	**Modified**	200	38	162	62	100	61.72%
**Vinca major L**.	**Traditional**	200	4	196	52	144	73.47%
	**Modified**	329	5	324	97	227	70.06%

**Figure 11:**
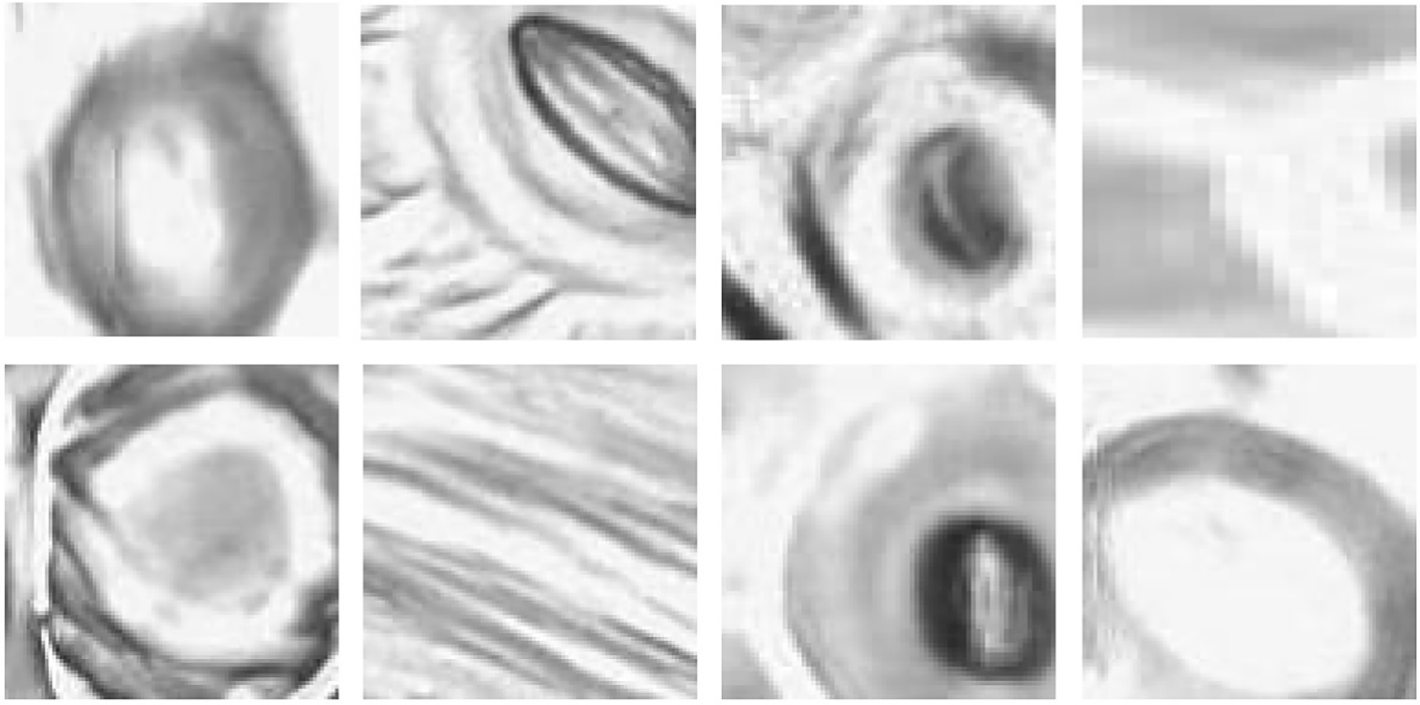
Examples of pores rejected by the pore estimation algorithm.

**Figure 12:**
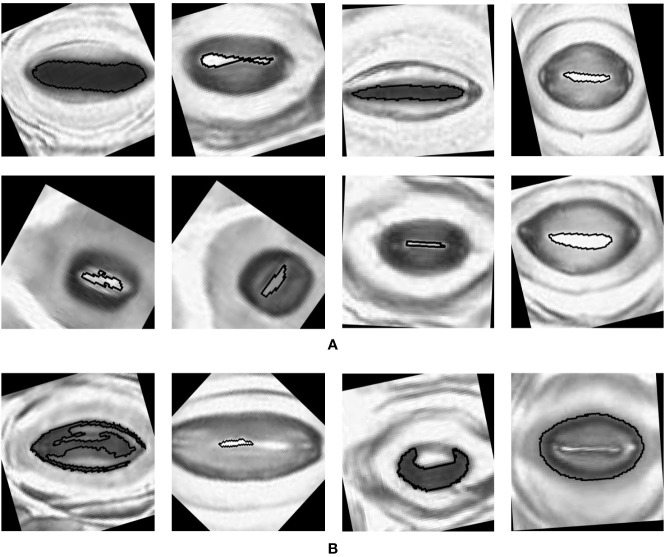
Examples of pores estimated by the algorithm **(A)** Correct estimations. **(B)** Incorrect estimations.

The pore estimation algorithm rejected 182 images where it was unable to make a confident prediction about the pore area (examples in **Figure 11**). The majority of the rejected images contained either false positives from the classifier or stomata where a pore was not visible. Out of the 1,557 images for which stomata pore areas were present, 409 were found to be estimating the pore incorrectly. A result is considered incorrect when the estimation has no overlap with the true pore region, or the pore boundaries of the estimation and the ground truth has a considerable mismatch. This results in an average pore estimation accuracy of 73.72% with a maximum pore estimation accuracy of 90.1%, achieved for Prunus armeniaca samples collected using the modified imprint method. The algorithm works well against stomata from different species, collected using both traditional and modified nail polish methods (**Figure 12**). In a research scenario, the erroneous results generated by the pore estimation algorithm can be easily identified and discarded via manual observation.

## Discussion

This research presents a practical pipeline to automatically assess stomatal number and aperture size with minimal human intervention. Key contributions were made in sample imaging and stomatal pore area calculation techniques, whilst existing sample collection and stomata detection methods were adopted and modified to optimize results. The final, end-to-end solution that begins with a plant leaf input, creates a high-quality digital representation, and automatically detects and measures stomata pores.

Currently, with traditional manual stage microscopes, imaging an average sized leaf sample (30 mm by 9 mm) would take multiple days. To alleviate this problem, we took inspiration from the field of cell pathology, where slide scanners are used to produce high-quality images of cell samples rapidly and automatically. This method dramatically reduced the imaging time, covering an entire 30 mm × 9 mm sample in 30–40 min. In addition, using the slide scanner offers great potential to image a large portion of a leaf with minimal human interaction. Unlike most current research, which analyses input images containing up to 40 stomata sampled at random locations on a leaf sample, this technique has shown accuracy on large, continuous sections of a leaf containing over 1,000 stomata. This can offer insights into the structure of leaves and the morphological properties they entail.

Upon digitization of the leaf samples, a CNN was used to detect stomata in the image, which effectively distinguished stomata from a highly detailed background containing visually similar guard cells. The average precision, recall, and F-score of 0.79, 0.85, and 0.82, respectively, indicate an approach that can be relied upon to accurately assess stomata.

Upon stomata detection, an approach which uses binary image segmentation and stomata cross section analysis was developed to accurately measure stomata pore areas. For the first time, an algorithm is developed, where the pore area can be detected despite the colour of the pore with respect to the surrounding guard cells. The proposed algorithm performed well, with an average pore estimation accuracy of 73.72% across 8 different collections. Although machine learning techniques are widely used for stomata detection, not many research projects tackle the problem of automatic pore measurement. In that context, the pore measurement methodology adds value to the process of fully automating stomata analysis.

The ability to rapidly and consistently assess the number and aperture of stomata over a relatively large portion of a leaf has a number of potential applications in plant science. Plants respond to the changing atmospheric carbon dioxide concentration by altering the ratio between the number of epidermal and stomatal cells (Beerling and Royer, 2002) and can offer a range of responses to increasing temperature; either increasing or decreasing stomatal size and density depending on conditions and species (Wu et al., 2018). Stomatal patchiness (Beyschlag and Eckstein, 2001), or the irregular distribution of stomata across a leaf, has received much attention in recent decades, but is yet to be completely understood. Once again, the slide scanner’s ability to rapidly image complete samples offers a valuable method for investigating this phenomenon on a large scale and would offer great benefits for investigators in this field. By investigating the area of open stomata relative to the leaf area, it may be possible to estimate stomatal conductance (Lawson et al., 1998). The pipeline is currently optimized to assess nail polish imprints, but as imaging systems improve; a system based on a field microscope may be developed to assess plant water stress and inform irrigation schedules.

## Data Availability Statement

The raw data supporting the conclusions of this article will be made available by the authors, without undue reservation.

## Author Contributions

LM developed the sample collection and imaging mechanisms, worked on stomata detection, and wrote the paper. HJ developed the stomata pore detection algorithm, worked on sample preparation, and wrote the paper. HP developed the Alexnet neural network. VK worked on stomata detection and image management. FT worked on sample preparation and imaging using the slide scanner. PP collected samples for the study and reviewed the paper. MW managed the overall project and reviewed the paper.

## Conflict of Interest

The authors declare that the research was conducted in the absence of any commercial or financial relationships that could be construed as a potential conflict of interest.
